# Comparative Histology of the Cornea and Palisades of Vogt in the Different Wild Ruminants (Bovidae, Camelidae, Cervidae, Giraffidae, Tragulidae)

**DOI:** 10.3390/ani12223188

**Published:** 2022-11-17

**Authors:** Joanna Klećkowska-Nawrot, Karolina Goździewska-Harłajczuk, Karolina Barszcz

**Affiliations:** 1 Department of Biostructure and Animal Physiology, Faculty of Veterinary Medicine, Wroclaw University of Environmental and Life Sciences, Kozuchowska 1, 51-631 Wroclaw, Poland; 2 Department of Morphological Sciences, Institute of Veterinary Medicine, Warsaw University of Life Sciences—SGGW, Nowoursynowska 159c, 02-787 Warsaw, Poland

**Keywords:** cornea, histology, palisades of Vogt, wild ruminants

## Abstract

**Simple Summary:**

In this study, for the first time, we performed a detailed histological analysis of the cornea and palisades of Vogt in the different wild ruminant species. The material for the research was taken from 49 adult wild ruminants (Bovidae, Camelidae, Cervidae, Giraffidae and Tragulidae) constituting 13 species coming from the Wroclaw Zoological Garden (Poland), the Warsaw Zoological Garden (Poland) and own collection of the Division of Animal Anatomy (Wroclaw). Our results showed that the number of layers of the cornea, i.e., five layers (anterior corneal epithelium, anterior limiting membrane (Bowman’s layer), the proper substance of the cornea, the posterior limiting membrane (Descemet’s membrane) and posterior corneal epithelium) or four layers (no Bowman’s layer) is not constant within even the same genus that includes the given species of the tested animal. The results of this study can form the basis for further research on the immunohistochemistry of the cornea in the maintenance of structural integrity and fluid balance in wild ruminants that can be used in veterinary ophthalmology diagnostics.

**Abstract:**

In the study, we data concerning the histological and morphometrical examination of the cornea and palisades of Vogt in the different species of ruminants from the families Bovidae, Camelidae, Cervidae, Giraffidae and Tragulidae, coming from the Warsaw Zoological Garden, the Wroclaw Zoological Garden and the Division of Animal Anatomy. The following ruminant species were investigated: common wildebeest, Kirk’s dik-dik, Natal red duiker, scimitar oryx, sitatunga, Philippine spotted deer, Père David’s deer, moose, reindeer, reticulated giraffe, okapi, Balabac mouse-deer and alpaca. The cornea of ruminant species such as the common wildebeest, Kirk’s dik-dik, Natal red duiker, scimitar oryx, reindeer and Balabac mouse-deer consisted of four layers (not found in the Bowman’s layer): the anterior corneal epithelium, the proper substance of the cornea, the posterior limiting membrane (Descemet’s membrane) and the posterior corneal epithelium (endothelium). The anterior corneal epithelium was composed of a multilayer keratinizing squamous epithelium, which was characterized in the studied ruminants with a variable number of cell layers but also with a different thickness both in the central epithelium part and in the peripheral part. Moreover, the proper substance of cornea was thinnest in Balabac mouse-deer, Kirk’s dik-dik, Natal red duiker, scimitar oryx, Philippine spotted deer, alpaca, reindeer and sitatunga and was thickest in the reticulated giraffe. The thickest Descemet’s membrane was observed in the Père David’s deer. The corneal limbus is characterized by a large number of pigment cell clusters in Kirk’s dik-dik, scimitar oryx, moose, Balabac mouse-deer and alpaca. In the common wildebeest, Père David’s deer, moose, reticulated giraffe, okapi and alpaca, the palisades of Vogt were marked in the form of a crypt-like structure. The corneal limbus epithelium in the examined ruminants was characterized by a variable number of cell layers but also a variable number of melanocytes located in different layers of this epithelium. The detailed knowledge of the corneal structure of domestic and wild animals can contribute to the even better development of methods for treating eye diseases in veterinary medicine.

## 1. Introduction

The cornea is a transparent and mechanical strength structure that covers the anterior part of the eyeball [1,2,3]. Together with the opaque sclera, it forms the fibrous membrane of the eyeball. Its diameter in humans is 1.0 – 11.5 mm on average [4]. A properly developed cornea is completely transparent and has a smooth surface, and its radius of curvature in humans is 7.5 – 7.8 mm on average [5]. The function of the cornea is to protect against injuries and the penetration of foreign bodies into the eye. Moreover, the cornea is an important structure of the optical system of the eye [6]. When the light rays pass through the cornea, they are refracted through it and properly focused on the retina for clear vision. Thanks to it, it is also possible to accommodate the eye, i.e., adjust the vision to different distances. The ciliary muscle is involved in this process. When we look at an object close to us, the muscle tightens, and the lens takes a round shape, thanks to which it has a greater ability to focus light and refract it. By staring at a distant object, the ciliary muscles relax, the lens is flattened, and the light is refracted less. If the cornea is no longer spherical and the refracted light in the vertical plane is not the same as in the horizontal plane, astigmatism (ataxia) will appear. It is a refractive error that leads to a deterioration of visual acuity and a decrease in contrast sensitivity [7,8,9].

The human cornea consists of five layers: the anterior stratified squamous non-keratinized epithelium, an anterior limiting membrane (Bowman’s layer) composed of irregularly spaced collagen fibers, about 8 – 14 µm thick, the proper substance of cornea with its keratocytes embedded in a hydrated matrix, the posterior limiting membrane (Descemet’s membrane) and the posterior corneal epithelium [4,10,11]. Bowman’s membrane is not a characteristic feature of all mammals and is absent in dogs, cats and lemurs [12]. Recent studies have shown, however, that in humans, there is another layer between the corneal core and the posterior border lamina—Dua’s layer (which was defined as a pre-Descemet’s membrane) [13,14]. Even though it is only 15 µm thick, it is really hard and durable (pressures of 1.5–2 bar are not a problem). The existence of Dua’s layer was confirmed during the simulation of a corneal transplant (deep anterior lamellar keratoplasty surgery). The procedure was performed on eyeballs from banks in Bristol and Manchester (Great Britain). To delicately separate the individual layers, fine air bubbles were injected into the cornea and then viewed under an electron microscope [13].

Ruiz-Ederra et al., [15] cited by Nautscher et al., [3] and Brunette et al. [16] report that species-specific differences in structural and physiological properties of the cornea are increasingly the focus of interest because animal corneal tissue is frequently used in human research and for therapeutic purposes. Therefore, basic histological knowledge of the corneal structures of domestic animals is mandatory.

Morphological evaluation of domestic and sparse wild ruminants cornea was described in the ox, the reindeer, the deer, the elk, the camel, the cow, the sheep, the sambar, the red deer, the giraffe, the zebu, the blackbuck, the mouflon, the eland and the mutton [3,17,18,19,20,21,22,23,24,25].

The corneal limbus is the boundary between the cornea and the sclera in which there are conjunctival folds called palisades of Vogt, containing niches for the limbal epithelial stem cell (LESC) [26]. The LESCs are responsible for the regeneration of the corneal surface and help maintain its transparency. These cells are found only in Vogt’s palisades, which create a special microenvironment for their renewal and proliferation. In humans, deficiency of these cells leads to corneal opacification through conjunctivalization and vascularization of the transparent cornea [27,28,29]. The LESC in humans deficit is influenced, in addition to by genetic diseases (aniridia, multiple endocrine deficiencies, erythrokeratodermia), also by chemical, heat or radiation burns, chronic inflammatory disease (Stevens-Johnson syndrome, ocular cicatricial pemphigoid, and infectious keratitis), contact lens-induced keratopathy, and iatrogenic multiple ocular surgeries [30]. However, in the case of domestic, especially wild mammals, the incidence of conjunctivalization in corneal disease is difficult to define because the methods that demonstrate the presence of conjunctivalization, such as impression cytology, are not commonly employed in veterinary ophthalmology [26].

Our research aimed to compare the histological structure and morphometry of individual layers of the cornea in different species of wild ruminants included in the five families constituting the Pecora infraorder and demonstrate revealing similarities and differences among this examined animals and to compare it to the domestic ruminants. This work can also be the basis for further research on the immunohistochemistry of the cornea in the maintenance of structural integrity and fluid balance in wild ruminants that can be used in veterinary ophthalmology diagnostics. In addition, knowledge of the morphology of the corneal limbus area in domestic and wild animals will improve ophthalmic procedures in veterinary medicine, which are limited due to the lack of data on the anatomy of this area of the limbus, the presumed presence of stem cells and their identification in various species of not only domestic animals but also and wild.

## 2. Materials and Methods

### 2.1. Collection of Specimen and Conservation Status

The material for the research was taken from 49 adult wild ruminants (Bovidae, Camelidae, Cervidae, Giraffidae, Tragulidae) constituting 13 species coming from the Wroclaw Zoological Garden (Wroclaw, Poland), the Warsaw Zoological Garden (Warsaw, Poland) and own collection of the Division of the Animal Anatomy (Wroclaw, Poland). The research was carried out on common wildebeest (*Connochaetes taurinus*), Kirk’s dik-dik (*Madoqua kirkii*), Natal red duiker (*Cephalophus natalensis*), scimitar oryx (*Oryx dammah*), sitatunga (*Tragelaphus spekii*), Philippine spotted deer (*Rusa alfredi*), Père David’s deer (*Elapharus davidanus*), moose (*Alces alces*), reindeer (*Rangifer tarandus*), reticulated giraffe (*Camelopardalis reticulate*), okapi (*Okapia johnstoni*), Balabac mouse-deer (*Tragulus nigricans*) and alpaca (*Vicugna vicugna*). These animals were collected from 2013 to 2022. The characteristics of the species of examined ruminants (status to the –International Union for Conservation of Nature (IUCN) Red List of Threatened Species (2022-1) [31], the number of specimens tested and the date of material collection) are given in Table 1.

### 2.2. Ethical Statement

According to Polish and European law, studies on tissues obtained post-mortem do not require the approval of the Ethics Committee (2010/63/EU Directive of the European Parliament and of the Council of 22 September 2010 on the protection of animals used for scientific purposes) and The Journal of Laws of the Republic of Poland, the Act of 15 January 2015, on the protection of animals used for scientific or educational purposes). Post-mortem animal material was obtained with personal permits issued by the District Veterinary Officer in Wroclaw (Poland) (No. PIW Wroc. UT-45/5/16—Dr. Joanna Klećkowska-Nawrot; No. PIW Wroc. UT-45/6)/16—Dr. Karolina Goździewska-Harłajczuk).

### 2.3. Histological Study

The eyeballs retrieved from all examined animals were placed in 4% buffered formaldehyde for at least 72 h and then rinsed in running water for 24 h. Then they were processed in a vacuum tissue processor—ETP (RVG3, Intelsint, Villarbasse, Italy) and embedded in paraffin. The specimens were cut using a Slide 2003 (Pfm A.g., Köln, Germany) sliding microtome into 4 µm sections. The hematoxylin & eosin and Picro-Mallory trichrome staining methods were applied. The slides obtained were then observed using the Zeiss Axio Scope A1 light microscope (Carl Zeiss, Jena, Germany) and were rated using scoring systems based on a standard protocol previously described [32,33]. NAV [34] and NHV [35] were used for the histological description of the examined structures. Histometric measurements of the corneal structures (anterior corneal epithelium, anterior limiting membrane, proper substance of cornea and posterior limiting membrane) were performed in the Axio Vision Rel. 4.8. (Carl Zeiss, Jena, Germany).

## 3. Results

**Figure 1:**
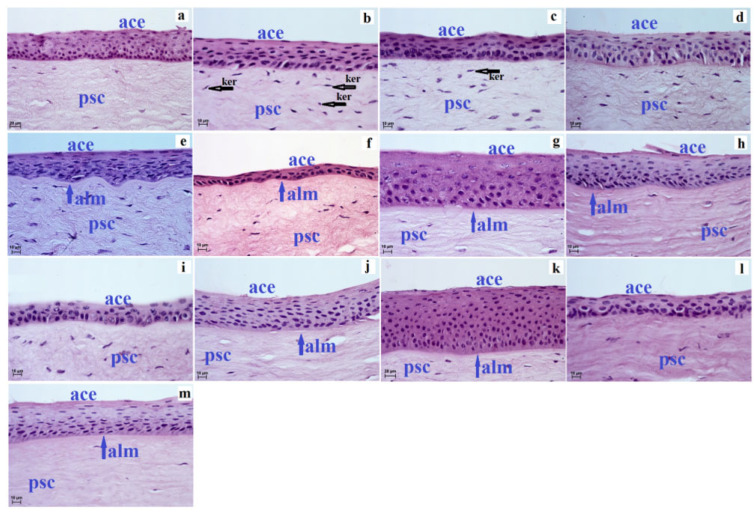
The photomicrograph of the cornea in the examined ruminants. H&E stain. (**a**)—common wildebeest, (**b**)—Kirk’s dik-dik, (**c**)—Natal red duiker, (**d**)—scimitar oryx, (**e**)—sitatunga, (**f**)—Philippine spotted deer, (**g**)—Père David’s deer, (**h**)—moose, (**i**)—reindeer, (**j**)—reticulated giraffe, (**k**)—okapi, (**l**)—Balabac mouse-deer, (**m**)—alpaca. Scale bar: (**a**,**k**) = 20 µm; (**b**–**j**,**l**–**m**) = 10 µm. ace—anterior corneal epithelium, alm—anterior limiting membrane (Bowman layer—blue arrow), ker—keratocytes (black arrow), psc—the proper substance of the cornea.

Our research showed that cornea in ruminant species such as common wildebeest, Kirk’s dik-dik, Natal red duiker, scimitar oryx, reindeer and Balabac mouse-deer consisted of four layers: anterior corneal epithelium, the proper substance of cornea, posterior limiting membrane (Descemet’s membrane) and posterior corneal epithelium (endothelium). However, in the sitatunga, Philippine spotted deer, Père David’s deer, moose, reticulated giraffe, okapi and alpaca, the cornea consisted of five layers because there was an anterior limiting membrane (Bowman’s layer) located between the anterior corneal epithelium and the proper substance of cornea (Figure 1).

The anterior corneal epithelium is the stratified squamous non-keratinized epithelium, which in examined ruminants was characterized by a variable number of cell layers in the central epithelium part: the lowest number of cell layers was in Philippine spotted deer (three to four) and Balabac mouse-deer (four to five), while the highest number of cell layers was found in the okapi (15–16), but the epithelium was also characterized by a variable number of cell layers in the peripheral epithelium part, where the lowest number of cell layers was found in Natal red duiker (three to four) and Balabac mouse-deer (three to four), while the highest number of cell layers was found in alpaca (13–14) and moose and okapi after 14–15 (Table 2). In addition, the anterior corneal epithelium was also characterized by different sizes in the central epithelium part and peripheral epithelium part in the examined ruminants (the thinnest anterior corneal epithelium was in the Philippine spotted deer 15.456 (±2.2) µm, the thickest was in okapi 130.315 (±7.6) µm in the central epithelium part, similarly in the peripheral epithelium part was also thinnest in Philippine spotted deer 16.891 (±2.7) µm, and thickest was in okapi 115.923 (±6.9) µm (Table 2). The cells of the wart were: superficial cells, intermediate cells and basal cells. The superficial cells were polygonal with flattened nuclei and clear cytoplasm, and where many of the cells underwent a desquamation process. The intermediate layers in the examined ruminants consist of cells with a wing-like appearance, where the cell nuclei were flattened in Kirk’s dik-dik, sitatunga, Philippine spotted deer and alpaca, while in the other studied species, the animals’ cell nuclei were large and round. The basal cells in all ruminants were isoprismatic/cylindrical with oval and big nuclei (Figure 1).

The Bowman’s layer in the examined ruminants was observed only in the sitatunga, Philippine spotted deer, Père David’s deer, moose, reticulated giraffe, okapi and alpaca, where Philippine spotted deer was the thinnest among these species, and its thickness was 2.5 ( ± 0.4) µm and in moose where its thickness was 3.286 ( ± 0.7) µm, and the thickest was at Père David’s deer where its thickness was 8.401 ( ± 1.1) µm (Table 3). The Bowman’s layer is made of thin collagen fibers.

**Figure 2:**
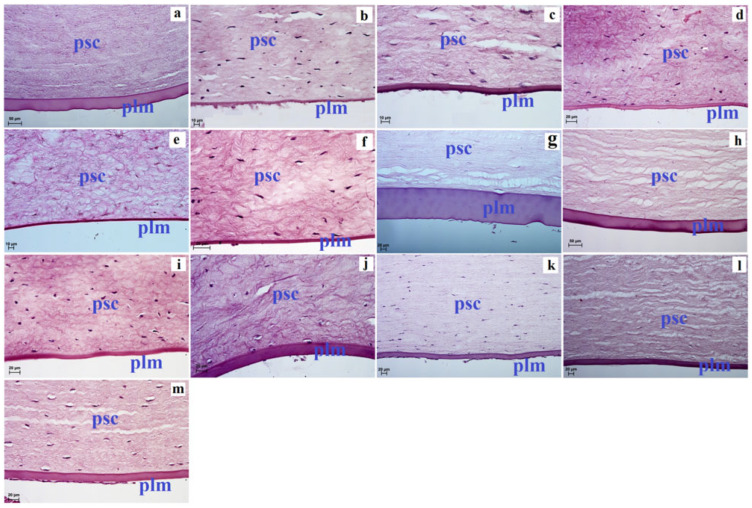
The photomicrograph of the cornea in the examined ruminants. H&E stain. (**a**)—common wildebeest, (**b**)—Kirk’s dik-dik, (**c**)—Natal red duiker, (**d**)—scimitar oryx, (**e**)—sitatunga, (**f**)—Philippine spotted deer, (**g**)—Père David’s deer, (**h**)—moose, (**i**)—reindeer, (**j**)—reticulated giraffe, (**k**)—okapi, (**l**)—Balabac mouse-deer, (**m**)—alpaca. Scale bar: (**a**,**h**) = 50 µm; (**d**,**f**–**m**) = 20 µm; (**b**,**c**,**e**) = 10 µm. plm—posterior limiting membrane (Descemet’s membrane), psc—proper substance of cornea.

The proper substance of the cornea is the thickest layer of the cornea in the tested animals (the thinnest was in Balabac mouse-deer, Kirk’s dik-dik, Natal red duiker, scimitar oryx, Philippine spotted deer, alpaca, reindeer and sitatunga, where it was in the range from 571.808 (±47.5) µm to 885.483 (±20.5) µm; intermediate values were observed in Père David’s deer, okapi and common wildebeest, where its thickness ranged from 1347.154 (±30.7) µm to 1461.735 (±57.7) µm, while the thickest the proper substance of cornea was in reticulated giraffe where its thickness was 1971.646 (±194.1) µm (Table 3). The corneal stroma was composed of a uniform collagen fibril matrix. Between this matrix of collagen fibers, the flattened and elongated keratocytes were observed (Figure 1 and Figure 2). Our studies have shown that the proper substance of cornea of the common wildebeest, Père David’s deer, moose and reticulated giraffes had very few keratocytes (Figure 1 and Figure 2).

The deep proper substance of cornea rested on the posterior limiting membrane, which was thinnest in Philippine spotted deer, scimitar oryx, Kirk’s dik-dik, sitatunga, reindeer and alpaca where the thickness of the Descemet’s membrane was in the range of 4.32 (±0.4) µm–8.097 (±0.6) µm; intermediate values were observed in alpaca, Balabac mouse-deer, reticulated giraffe, okapi, common wildebeest and moose, where the thickness of this membrane ranged from 4.922 (±0.7) µm–47.366 (±2.3) µm, while the thickest was in Père David’s deer 118.571 (±3.3) µm (Figure 2 and Table 3).

The posterior surface of the cornea was a single-layer squamous epithelium, also called the posterior corneal epithelium or the endothelium of the anterior chamber of the eyeball.

**Figure 3:**
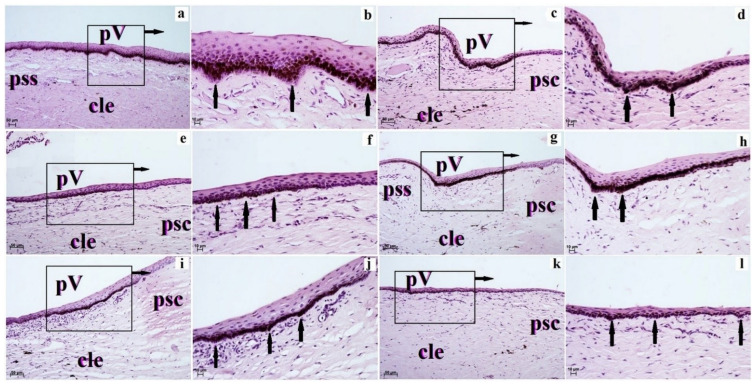
The photomicrograph of the palisades of Vogt in the examined ruminants. H&E stain. (**a**,**b**)—common wildebeest; (**c**,**d**)—Kirk’s dik-dik; (**e**,**f**)—Natal red duiker; (**g**,**h**)—scimitar oryx; (**i**,**j**)—sitatunga; (**k**,**l**)—Philippine spotted deer. Scale bar: (**a**,**c**,**e**,**g**,**i**,**k**) = 50 µm; (**b**,**d**,**f**,**h**,**j**,**l**) = 10 µm. cle—corneal limbus epithelium, psc—the proper substance of the cornea, pss—the proper substance of the sclera, pV—palisades of Vogt (black arrows).

**Figure 4:**
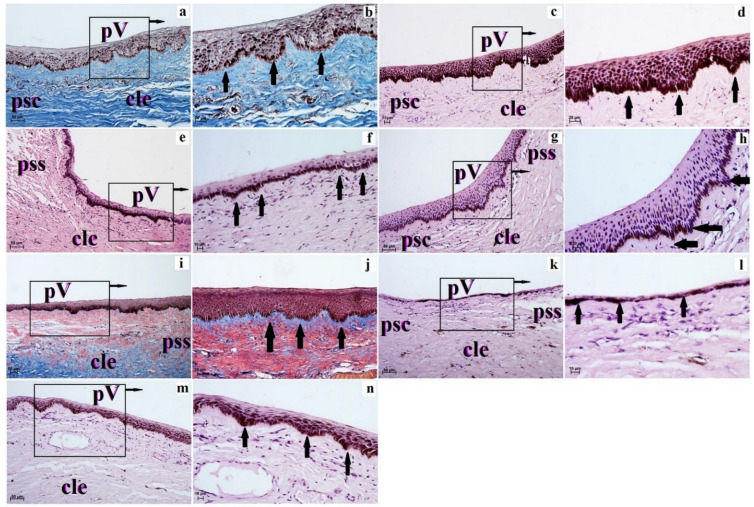
The photomicrograph of the palisades of Vogt in the examined ruminants. H&E stain (**c**–**h**,**k**–**n**); Picro-Mallory stain (**a**,**b**,**i**,**j**). (**a**,**b**)—Père David’s deer; (**c**,**d**)—moose; (**e**,**f**)—reindeer; (**g**,**h**)—reticulated giraffe; (**i**,**j**)—okapi; (**k**,**l**)—Balabac mouse-deer; (**m**,**n**)—alpaca. Scale bar: (**a**,**c**,**e**,**g**,**i**,**k**,**m**) = 50 µm; (**b**,**d**,**j**,**h**) = 20 µm; (**f**,**l**,**n**) = 10 µm. cle—corneal limbus epithelium, psc—the proper substance of the cornea, pss—the proper substance of the sclera, pV—palisades of Vogt (black arrows).

The corneal limbus was located at the junction of the cornea and sclera and characterized by the loss of the anterior limiting membrane (examined ruminants with Bowman’s layer in those species) and organization of the collagen fibers with large numbers of pigment cells present in Kirk’s dik-dik, scimitar oryx, moose, Balabac mouse-deer and alpaca. Pod corneal limbus epithelium within the superficial stroma of corneal limbus was observed in blood and lymphatic vessels (Figure 3 and Figure 4).

The corneal limbus epithelium in the examined animals was composed of a different number of cell layers (Table 4). The smallest number of cell layers forming corneal limbus epithelium was in Balabac mouse-deer (3–4) and Philippine spotted deer (4–6), while the largest number of cell layers was in the sitatunga (15–19–20), reticulated giraffe (17–18) and okapi (18–19; Table 4). The superficial layer was composed of squamous epithelium flattened nuclei, intermediate layers to wing cells with an oval nucleus, and a layer of basal cells with cylindrical shape (Natal red duiker, scimitar oryx, sitatunga, Philippine spotted deer, moose, reticulated giraffe and okapi) or is prismatic shape (common wildebeest, Kirk’s dik-dik, Père David’s deer, reindeer, Balabac mouse-deer and alpaca) with round nuclei (Figure 3 and Figure 4). Moreover, in the examined ruminants (except alpaca—no melanocytes in all cell layers), the corneal limbus epithelium is characterized by a variable amount of melanocytes located in different layers of the epithelium (Table 4, Figure 3 and Figure 4).

## 4. Discussion

The cornea is primarily a protective eye (resistance against external hazards) but also plays an important role in the optical system of the eye, which is primarily influenced by its transparency as well as its ability to refract and focus light rays. Most of the research on the structure and function of the cornea to create more and more perfect methods of treating the diseases of the cornea itself is most often conducted on laboratory animals (mice, rats, rabbits, and hens) which are animal models for human resources [36,37,38,39,40,41]. Corneal morphology studies in domestic animals, such as dogs, cats, equine, porcine, cows, goats or sheep, which also become animal models [3,12,17,42,43,44,45,46,47]. However, there are few publications on a comparative morphological examination of the cornea in wild animals, including ruminants.

The present study demonstrated a multilayered cornea, which was composed in all examined animals of the stratified squamous non-keratinized epithelium, the proper substance of cornea with an attached posterior limiting membrane (Descemet’s membrane) and posterior corneal epithelium. On the other hand, the situation with the presence or absence of an anterior limiting membrane, also known as Bowman’s layer, located under the anterior corneal epithelium is interesting. Our research showed a clear presence of Bowman’s layer in the sitatunga, Philippine spotted deer, Père David’s deer, moose, reticulated giraffe, okapi and alpaca, but no such presence in the field of view of common wildebeest, Kirk’s dik-dik, Natal red duiker, scimitar oryx, reindeer and Balabac mouse-deer. Bowman’s layer was first described in humans by William Bowman in 1947 [48], quoted by Merindano et al., [12] whereas a 9.7 ± 1.7 µm thick, acellular structure that consists of three to four collagen layers [49] cited by Nautscher et al., [3]. Mindanao et al. [12], in a study on 40 different species of mammals (Carnivores, Primates and Herbivores), showed that Bowman’s layer does not occur in Carnivores, *Lepilemur mustelinus* (Primates) (similarly to *Macaca mulata Wislocki*, [50]) as well as in *Tapirus terrestris, Equus caballus, Equus caballus przewalskii, Cervus elaphus, Rangifer tarandus, Antilope cervicapra, Ovis musimon, Ovis aries, Sus Domestica, Sus scrofa* and *Loxodonta africana*. However, regarding only ruminants, according to Merindano et al. [12], This layer occurred in *Dama dama, Cervus unicolor, Giraffa camelopardalis, Bos primigenius, Bos indicus* and *Taurotragus oryx*. Comparing our research and that of Merindano et al. [12], it can be seen that the presence or absence of the Bowman’s layer is a species feature. According to Nautscher et al., [3] his research showed that this layer does not occur in domestic animals (pig, cow, goat, horse, dog and cat); however, there is a disagreement about the existence of a Bowman’s layer in domestic animals. However, the above-mentioned authors suggest that this layer is not developed in domestic animals to a similar dimension as humans and other primates. However, Cafaro et al. [17] report that in the merino sheep occurs a relatively thin Bowman’s layer. It is, therefore, worth explaining in the future to clarify this morphological detail using electron microscopic methods, but it is also interesting that some electron and in vivo confocal microscopy studies in dogs and horses also failed to address the existence of a specific Bowman’s layer within the corneal layers [51,52]. Moreover, our research and the research of Merindano et al. [12] showed that Bowman’s layer in examined ruminants had a different thickness between different species, which may indicate a species characteristic of animals. However, the explanation in detail as to whether the presence of Bowman’s layer gives any evolutionary advantage to the different species needs future studies.

The anterior corneal epithelium in our examination of ruminants showed clear variations in the size of the epithelium (thick) both in the central and peripheral epithelium part and also a different number of cell layers between different animal species. High variations in the number of cell layers were found, especially in the superficial and intermediate layers. Also, large differences in their research were presented in some wild ruminants by Merindano et al. [21], where there were significant differences between animal species both in the thickness of the anterior corneal epithelium but also in the number of cell layers and the central part and peripheral part of this epithelium. Nautscher et al. [3] demonstrated in their studies that the number of cell rows correlates with the corneal thickness, while the thickness of the cornea changes with the age of the subject [53]. Nautscher et al. [3] also report that the physiological reason for the different number of cell layers in the anterior corneal epithelium remains unclear, but they suggest that corneal thickness is related to the habitat and environment of animals (the herbivores living in open grasslands are more likely exposed to rough environmental conditions than carnivores) [1,42]. Almubrad and Akhtar [1], in a study carried out on camels, showed that the anterior corneal epithelium was extraordinarily thick, which was justified by the fact that they attributed it to the hot and dry climate where camels normally live. By analyzing our corneal epithelial measurements and comparing them with those performed in domestic animals by Nautscher et al. [3], we find that in pigs, cows, goats, horses, dogs and cats, this epithelium was thickness than our ruminants tested. In addition, our research also showed large differences in the thickness of the proper substance of the cornea and Descemet’s membrane between the investigated ruminant species.

At the junction of the cornea and sclera, there is a corneal limbus, which has meridian-oriented conjunctival folds known as Vogt palisades. Vogt’s palisades contain niches for corneal limbal stem cells, which can regenerate the cornea [54,55]. According to Patruno et al. [55], corneal limbal stem cell therapies in veterinary medicine are limited due to the lacking of knowledge about the anatomy of the limbal area, the putative presence of stem cells and their identification in domestic species. Schermer et al., [56] cited by Patruno et al., [55] proposed that the corneal epithelial stem cells are not uniformly distributed throughout the entire corneal epithelial basal layer but are preferentially located in the limbal epithelial basal layer and the basal layer of the limbus was described as the corneal stem cells niche [57]. The corneal limbal stem cells are responsible for the regeneration of the corneal surface and are involved in maintaining its transparency. In the case of their insufficiency, usually caused by deficiency or damage, the cornea is covered with the conjunctival epithelium, which leads to its cloudiness and the formation of a vascularized endosperm. Such a situation often occurs after severe burns of the eyeball or in the case of congenital diseases such as keratitis ichthyosis deafness (KID) syndrome or aniridia, but it can also be an iatrogenic cause [58]. The corneal limbus epithelium was composed of the three cell layers (the superficial cells, intermediate cells and basal cells) in all examined ruminants as well as in domestic animals [55]. Our research and the research of Patruno et al. [55] showed that the epithelium was characterized by a variable total (total) number of cell layers in the tested animals, but also by the number of individual cell layers. The most interesting were basal cell alleys, where it turned out in our research that the cells that make up this layer, depending on the ruminant species, may take a typical cylindrical or isoprismatic shape. Similarly, in their studies on domestic animals, Patruno et al. [55] observed that in cats and dogs, the basal layer cells were cubic in shape as opposed to pigs, cows, sheep and horses, where they were cylindrical in shape. The situation is similar in the case of pigment accumulation within the individual three layers of the corneal limbus epithelium. Here, too, we observed differences between the ruminant species studied, where melanin was either located in all three cell layers, in only one or two layers, or there was no pigment vogue. Again, similar results were observed in domestic animals by Patruno et al. [55]. Another characteristic feature of our research and in the studies of Patruno et al. [55] we observed the presence of clearly marked crypt-like structures within the corneal limbus epithelium, or their absence or insignificant presence.

## 5. Conclusions

Our research and studies of other authors carried out on domestic animals as well as on a few species of wild animals showed that the number of layers of the cornea, i.e., 5 layers (anterior corneal epithelium, anterior limiting membrane (Bowman’s layer), proper substance of cornea, posterior limiting membrane (Descemet’s membrane) and posterior corneal epithelium) or 4 layers (no Bowman’s layer) is not constant within even the same genus that includes the given species of the tested animal. Moreover, it was observed that the thickness of individual layers of the cornea is also variable, but also the number of layers of cells forming the anterior corneal epithelium between given species, which may already constitute an individual feature. The presence of palisades of Vogt and limbal epithelial stem cells gathered there is an important feature of the cornea, not only in humans and domestic animals but also in wild animals, which in the case of wild animals requires further and very detailed research because it should be assumed that the specific habitat of such animals also requires corneal surface regeneration. It is also interesting whether wild animals have a very specific Dua’s layer—the answer to this question requires a great deal of commitment from histologists, veterinary ophthalmologists as well as biologists using numerous research techniques. Summing up, getting to know the detailed structure of the cornea in domestic and wild animals can contribute to the even better development of methods of treating eye diseases in veterinary medicine.

## Figures and Tables

**Table 1:** Characteristics of the examined ruminants.

Infraorder	Family	Subfamily	Tribus	Genus	Species/ Subspecies	IUCN (2022-1)	Number of Collection/ Date of Collection	Source of Collection
Pecora	Bovidae	Antilopinae	Alcelaphini	*Connochaetes*	common wildebeest *Connochaetes taurinus*	LC stable	2 /2017	Wroclaw Zoological Garden
			Antilopini	*Madoqua*	Kirk’s dik-dik *Madoqua kirkii*	LC stable	7 /2016, 2017, 2018, 2019, 2021	Wroclaw Zoological Garden
			Cephalophini	*Cephalophus*	Natal red duiker *Cephalophus natalensis*	LC decreasing	6 /2016, 2017, 2020, 2021	Wroclaw Zoological Garden
			Hippotragini	*Oryx*	scimitar oryx *Oryx dammah*	EW	2 /2017	Wroclaw Zoological Garden
		Bovinae	Tragelaphini	*Tragelaphus*	sitatunga *Tragelaphus spekii*	LC decreasing	11 /2016, 2017, 2018, 2019	Wroclaw Zoological Garden
	Cervidae	Cervinae	Cervini	*Rusa*	Philippine spotted deer *Rusa alfredi*	EN decreasing	1 /2016	Wroclaw Zoological Garden
				*Elaphurus*	Père David’s deer *Elaphurus davidianus*	EW	1 /2017	Wroclaw Zoological Garden
		Capreolinae	Alceini	*Alces*	moose *Alces alces*	LC increasing	1 /2017	Warsaw Zoological Garden
			Odocoileini	*Rangifer*	reindeer *Rangifer tarandus*	VU decreasing	3 /2018, 2019	Wroclaw Zoological Garden
	Giraffidae	Giraffinae	–	*Giraffa*	reticulated giraffe *Giraffa Camelopardalis* *reticulata*	VU decreasing	1 /2016	Wroclaw Zoological Garden
			–	*Okapia*	okapi *Okapia johnstoni*	EN decreasing	1 /2017	Wroclaw Zoological Garden
Tragulina	Tragulidae	–	–	*Tragulus*	Balabac mouse-deer *Tragulus nigricans*	EN decreasing	8 /2016, 2018, 2019, 2022	Wroclaw Zoological Garden
Cameliformes	Camelidae	–	*Lamini*	*Vicunia* *pacos*	alpaca *Vicugna pacos*	LC MD increasing	6 /2013, 2014	Wroclaw Zoological Garden, Own collection of Division of Animal Anatomy

EN—endangered, EW—extinct in the wild, MD—moderately depleted, LC—last concern, VU—vulnerable.

**Table 2:** Morphometrical features of the anterior corneal epithelium in the examined ruminants.

	Anterior Corneal Epithelium									
	Number of Cellular Layers in the Central Cornea	Number of the Superficial Cell Layers	Number of the Intermediate Cell Layers	Number of the Basal Cell Layers	Thickness of the Central Part	Number of Cellular Layers in the Peripheral Cornea	Number of the Superficial Cell Layers	Number of the Intermediate Cell Layers	Number of the Basal Cell Layers	Thickness of the Peripheral Part
common wildebeest	8 – 9	3	4 – 5	1	47.493 ( ± 4.9)	7 – 8	1 – 2	5	1	35.948 ( ± 4.1)
Kirk’s dik-dik	6 – 7	2 – 3	3	1	28.873 ( ± 3.2)	6 – 7	2 – 3	3	1	29.596 ( ± 4.6)
Natal red duiker	5 – 6	1	3	2	35.208 ( ± 3.3)	3 – 4	1	1 – 2	1	19.255 ( ± 2.2)
scimitar oryx	5 – 6	2 – 3	2	1	35.606 ( ± 3.2)	5 – 6	2 – 3	2	1	35.577 ( ± 4.4)
sitatunga	8 – 9	3 – 4	2 – 5	1	51.218 ( ± 5.7)	9 – 10	1	6–7	3	42.712 (±4.2)
Philippine spotted deer	3 – 4	1 – 3	1	1	15.456 ( ± 2.2)	6 – 7	3	1 – 2	1 – 2	16.891 ( ± 2.7)
Père David’s deer	12 – 13	1 – 2	7 – 8	2 – 3	80.99 ( ± 1.8)	11 – 12	1 – 2	6 – 7	2 – 3	74.163 ( ± 3.7)
moose	10 – 11	1 – 2	7 – 8	1	42.073 ( ± 1.3)	14 – 15	1 – 3	12	1 – 2	75.63 ( ± 3.0)
reindeer	7 – 8	1 – 2	5 – 6	1	33.461 ( ± 3.1)	4 – 5	1	2 – 3	1	21.712 ( ± 3.4)
reticulated giraffe	9 – 10	2 – 3	6 – 7	1	42.93 ( ± 3.1)	10 – 11	2 – 3	6 – 7	2	42.072 ( ± 3.4)
okapi	15 – 16	2	11 – 12	1 – 2	130.315 ( ± 7.6)	14 – 15	2–3	10	2	115.923 ( ± 6.9)
Balabac mouse-deer	4 – 5	1 – 2	2	1	24.778 ( ± 3.8)	3 – 4	1	1 – 2	1	13.127 ( ± 0.9)
alpaca	7 – 8	2 – 3	4 – 5	1	50.563 ( ± 3.5)	13 – 14	1 – 2	9	2 – 3	55.651 ( ± 4.1)

**Table 3:** Thickness (µm) of the Bowman’s layer, the proper substance of the cornea and Descemet’s membrane in the examined ruminants.

Species	Bowman’s Layer	Proper Substance of Cornea	Descemet’s Membrane
common wildebeest	–	1461.735 (±57.7)	43.181 (±2.3)
Kirk’s dik-dik	–	651.772 (±19.4)	4.563 (±0.8)
Natal red duiker	–	669.987 (±34.9)	8.097 (±0.6)
scimitar oryx	–	697.172 (±17.7)	4.487 (±1.1)
sitatunga	4.04 (±0.5)	885.483 (±20.5)	5.028 (±0.8)
Philippine spotted deer	2.5 (±0.4)	645.331 (±14.6)	4.32 (±0.4)
Père David’s deer	8.401 (±1.1)	1347.154 (±30.7)	118.571 (±3.3)
moose	3.286 (±0.7)	1668.633 (±36.5)	47.366 (±2.3)
reindeer	–	869.367 (±33.1)	7.14 (±0.6)
reticulated giraffe	5.055 (±0.7)	1971.645 (±194.1)	20.387 (±2.1)
okapi	4.691 (±0.9)	1417.026 (±46.1)	27.661 (±1.3)
Balabac mouse-deer	–	571.808 (±47.5)	19.458 (±1.2)
alpaca	4.295 (±1.1)	679.217 (±19.2)	14.922 (±0.7)

**Table 4:** Characteristic of the corneal limbus epithelium (palisades of Vogt) in the examined ruminants.

	Corneal Limbus Epithelium				
	Total Number of Cell Layers	Number of Superficial Cell Layers	Number of Intermediate Cell Layers	Number of Basal Cell Layers	Obecność Melanocytes
common wildebeest	15–16	3	3–4–6	6–7	+++ (basal cell layers)
Kirk’s dik-dik	7–8	3	2–3	2–3	+ (all cell layers)
Natal red duiker	7–9	1–2	4	2–3	+ (basal cell layers)
scimitar oryx	7–8	2–3	3	2	++ (all cell layers)
sitatunga	15–19–20	1	13–16	1–2–3	+ (all cell layers)
Philippine spotted deer	4–6	1–2	2–3	1	−/+ (all cell layers)
Père David’s deer	17–18	3–4	12–13	1–2	++ (intermediate and basal cell layers)
moose	17 –18	2–3	13	2	+++ (all cell layers)
reindeer	8–9	1	5–6	2	+ (basal cell layers)
reticulated giraffe	17–18	2	14–15	1–2	−/+ (intermediate and basal cell layers)
okapi	19–20	1–2	13–16	1–2	−/+ (basal cell layers)
Balabac mouse-deer	3–4	1	1	1–2	+ (all cell layers)
alpaca	13–14	1	10–11	2–3	–

## Data Availability

Material available by request to the corresponding authors (karolina.gozdziewska-harlajczuk@upwr.edu.pl; joanna.kleckowska-nawrot@upwr.edu.pl).
